# A Review on Si Uptake and Transport System

**DOI:** 10.3390/plants8040081

**Published:** 2019-03-29

**Authors:** Harmanjit Kaur, Maria Greger

**Affiliations:** Department of Ecology, Environment and Plant Sciences Stockholm University, 10691 Stockholm, Sweden; harmanjit.kaur@su.se

**Keywords:** Si transporters, Si, monocots, eudicots, cryptogams, *Lsi1*, *Lsi2*, *Lsi6*

## Abstract

Silicon (Si) was long listed as a non-essential component for plant growth and development because of its universal availability. However, there has been a resurgence of interest in studying the underlying uptake and transport mechanism of silicon in plants because of the reported dynamic role of silicon in plants under stressed environmental conditions. This uptake and transport mechanism is greatly dependent upon the uptake ability of the plant’s roots. Plant roots absorb Si in the form of silicic acid from the soil solution, and it is moved through different parts of the plant using various influx and efflux transporters. Both these influx and efflux transporters are mostly found in the plasma membrane; however, their location and pattern of expression varies among different plants. The assessment of these features provides a new understanding of different species-dependent Si accumulations, which have been studied in monocots but are poorly understood in other plant groups. Therefore, the present review provides insight into the most recent research exploring the use of Si transporters in angiosperms and cryptogams. This paper presents an extensive representation of data from different families of angiosperms, including monocots and eudicots. Eudicots (previously referred to as dicots) have often been neglected in the literature, because they are categorized as low/intermediate Si accumulators. However, in this review, we attempt to highlight the accumulating species of different plant groups in which Si uptake is mediated through transporters.

## 1. Introduction

Silicon holds a unique position in plant biology because of its relative abundance (28.8%) in the Earth’s crust [1]. Though, not deemed to be a vital element for most plants, its uptake is associated with a number of benefits impacting normal growth and development. Crop plants, such as rice and sugarcane, are sometimes supplemented with Si fertilizers to enhance plant yield and crop quality [2,3]. The benefits of Si include resistance to biotic and abiotic stresses, such as drought, low temperature, salinity, metal toxicity, diseases and pests, enhancement of secondary metabolites in various medicinal plants, nutrient imbalances, and stimulation of photosynthesis [4,5,6]. The essentiality of Si has always been a topic of debate, as Si does not alter the normal growth and development of plants. Nevertheless, debates regarding essentiality must consider that food products derived from Si-exposed plants offer greater bone strength and improved nervous and immune system functions in human beings [7]. However, the beneficial effects of Si are greatly hampered by the absorption ability of plants, which, in turn, results in the arbitrary classification of plants as low accumulators (<0.1% Si), intermediary accumulators (1%), and high accumulators (more than 5% Si on dry weight basis) with respect to Si absorption [8]. For instance, rice, which is a monocot, accumulates up to 10% Si in shoots, whereas cucumber, a eudicot, is an intermediate accumulator that accumulates less Si than rice, and tomato, which is also a eudicot, is an extremely low-accumulating plant [9]. Therefore, in general, it is believed that monocots are high Si accumulators compared with eudicots. Different plants of the monocot families, such as Poaceae and Cyperaceae, accumulate more Si (>4%) than eudicot clades, such as Urticaceae and Cucurbitaceae, which accumulate 2–4% Si [10,11]. Table 1 shows different plant species with high, low, and intermediate degrees of Si accumulation in different groups of plants.

Therefore, efforts were made in this paper to corroborate the uptake of Si in different plant groups. Collective data representation was developed for Si transporters in angiosperms (monocots and eudicots) and cryptogams. Here, we focused on the channels/transporters responsible for Si uptake and transport in different groups of plants.

## 2. Mode of Cellular Si Uptake and Accumulation 

Si content varies greatly in different plant tissues, as it undergoes several chemical transformations during the storage, deposition and transport phases. More often, it is also due to the differences in the uptake and transport mechanism, which takes place in plants [13,14]. Three different types of water-based Si uptake mechanisms have been anticipated based upon the Si content in plants and their uptake rates relative to water [15,16]. Plants having higher Si uptake levels than water are classified as active (e.g., rice, wheat, and barley), whereas those with similar rates are classified as passive (e.g., oat) and those with lower uptake rates are classified as rejective. The uptake mechanism of Si requires specific transmembrane proteins identified as Si transporters to mediate the whole process [17]. These protein channels are passive transporters, which do not require energy to transport silicic acid across the plasma membrane. Some examples of plants with energy-dependent Si transport include rice, barley, maize, wheat, banana, and cucumber [18].

Si is transported from roots to shoots as silicic acid [Si (OH)_4_], which at biological pH has the ability to move across the plasma membrane [19]. In a comparative analysis of high, medium, and low Si-accumulating plants such as rice, cucumber, and tomato, it was observed that Si transport is facilitated by transporters (Km value 0.15 mM) across the cortical cells, whereas Vmax value followed a decreasing trend from rice to cucumber to tomato, thereby showing a significant variation in the transport density. Following primary uptake from the roots, Si is further transported to shoots via xylem. In shoots, silicic acid is polymerized into silica gel due to loss of water (transpiration) and is usually deposited in different plant parts [20]. For example, in rice, Si is deposited as a dense layer (2.5 μm) beneath the cuticle layer (0.1 μm), thus acting as a first line of defense against various kinds of stresses [21]. It is also deposited in bulliform cells of the leaf epidermis of durum wheat and forms a 1–4 μm thick layer, which provides mechanical support and protection [22]. 

## 3. Si Facilitation via Transporter Genes

### 3.1. Angiosperms 

#### 3.1.1. In Monocots

The main reservoir of Si (50–70%) in the form of Si dioxide is in the solid phase of soil. However, its availability to plants is relatively low. The mechanism of uptake of Si varies amongst different plant species and apparently depends on the presence of specific Si transporters. The very first report of the identification of Si transporters in plants was in rice [23]. More recently, transporters were also exposed in other plants including barley, wheat, and pumpkin [24,25]. The discovery of such evidence in rice, which suggests that the uptake mechanism of Si is quite active and even faster than that of water, made it quite clear that a specific system exists in rice that facilitates the uptake of Si. This led to the identification and cloning of *Lsi1* genes using a defective rice mutant, which was believed to be responsible for Si uptake in rice [26]. Transporter genes were localized on chromosome 2, with cDNA of 1409 bp consisting of 5 exons and 4 introns. Blast and ClustalW analysis showed that this transporter belongs to a NIP3 major protein family and an aquaporins subfamily [27]. When present in the plasma membrane, this transporter facilitates Si uptake into the root stele, which is otherwise prevented by the presence of casparian strips. *Lsi1* is expressed in the main and lateral roots but not in the root hairs, thereby justifying a previously reported study that found that root hairs lack participation in the Si uptake process [28]. Also, when cRNA-encoded *Lsi1* was inserted into a *Xenopus laevis* oocyte, it was seen that silicic acid transport activity increased significantly. All these findings confirm that *Lsi1* is a major influx transporter of Si into rice roots. 

**Figure 1:**
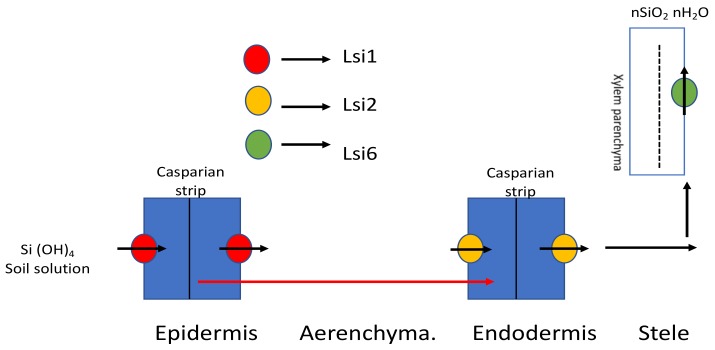
Flow of Si across the plant using Si transporters in rice. *Lsi1*, the influx transporter gene localized in the plasma membrane, is accountable for Si uptake into the exodermis of the cell; *Lsi2*, an efflux transporter, moves Si into the apoplast across the aerenchyma; *Lsi6* further takes it up the aerial parts of the plant. Modified with reference [32].

*Lsi2*, belonging to an anion transport family, facilitates Si uptake from the roots to the vascular tissues in rice [29,30]. *Lsi2* shares location similarity with *Lsi1* but lies towards the proximal side of the membrane, building up in the mature root zone instead of the root tips. Moreover, with injection into *Xenopus* oocytes, *Lsi2* showed completely efflux activity rather than any influx activity for silicic acid. Another Si transporter *Lsi6*, which moves silicic acid across the vascular bundle, has been identified in rice [31]. *Lsi6* is expressed in the parenchyma cells of the leaves. The absence of *Lsi6* has no negative effect on Si uptake except that it can lead to excess silicified epidermal depositions and also outburst the flow into the guttation fluid [32]. Si uptake and transport using Si transporters is depicted in Figure 1.

Influx transporter (*Lsi1*) takes silicic acid from soil solution up to the exodermis, followed by the efflux transporter (*Lsi2*), which takes it further across the aerenchyma. Then, it is further moved up the aerial parts of the plants by another influx transporter, *Lsi6* [32].

*Erysiphe graminis* f.sp. *tritici* causes powdery mildew in wheat, which is inhibited by the addition of Si to the wheat nutrient solution. This finding led to the discovery of *TaLsi1*, an influx transporter of Si [25,33]. This transporter was found to be quite similar to the *OsLsi1* influx transporter in rice in terms of localization and activity in oocytes. In another study, discovery of the *HvLsi1* influx transporter was made in barley; it was located in the distal side of the plasma membrane and showed 81% similarity with rice influx transporter, *OsLsi1* [34]. Despite such a high degree of similarity, it differed in terms of expression patterns. Maize (*Zea mays* L.) is a high Si accumulator crop plant, in which Si uptake is mediated by two genes *ZmLsi1* and *ZmLsi6*. *ZmLsi1* is located in the roots, while *ZmLsi6* is located in the parenchyma cells of the leaves. When expressed in the *Xenopus laevis* oocyte, both showed higher levels of permeability towards silicic acid. 

**Figure 2:**
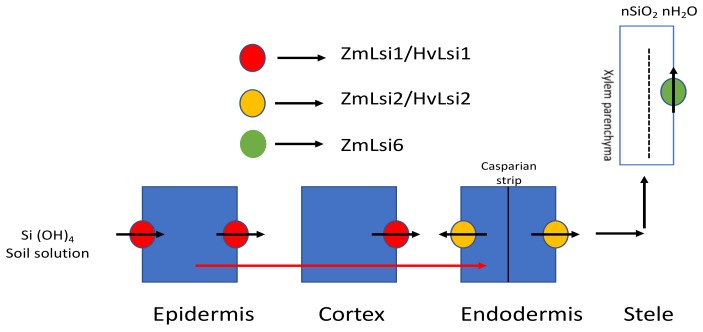
Si transporter-mediated Si transport in maize and barley. In maize and barley, Si is absorbed from the external solution using *ZmLsi1*/*HvLsi1* and transported further into the root cells from which, through the symplastic pathway, it is passed into the endodermis and further to the stele. *ZmLsi6* mainly functions as a Si transporter for xylem unloading. Modified with reference [32].

*OsLsi2* efflux homologues have also been found in barley (*HvLsi2*) and maize (*ZmLsi2*), although they have more than 80% similarity with *Lsi2* but differ in terms of localization, as both are present only in the endodermis and do not show polar localization [32]. *HvLsi2* and *ZmLsi2* also share functional similarity with *OsLsi2* in terms of activity and expression patterns. In corn, *ZmLsi6*, an influx Si transporter similar to *OsLsi6*, has also been isolated and characterized [35]. *ZmLsi6* is mainly responsible for xylem unloading and is localized in the parenchyma cells of xylem. There is a disparity in the uptake system between different plants, which is attributed to different root structures. In rice roots, there are two casparian strips at the exodermis and endodermis, whereas in maize and barley roots there is one casparian strip at the endodermis. Figure 2 shows the Si uptake mechanism mediated by different transporters in maize and barley. 

#### 3.1.2. In Eudicots

Analysis of the literature reveals that little information is available about Si transporters in eudicots, except with regard to a few members of the Cucurbitaceae, Urticaceae, and Asteraceae families, which show additional benefits with Si applications [11,24]. In pumpkin (*Cucurbita moschata*), *CmLsi1* was the first Si influx gene found in eudicots [24]. Two different pumpkin cultivars (bloom and bloomless *Cucumis sativus*) were studied for Si uptake activity and expression patterns. Heterogeneous expression in the *Xenopus* oocyte showed enhanced Si influx activity in the rootstock from the bloom pumpkin (*CmLsi1*B^+^), whereas no activity was reported in the bloomless rootstock (*CmLsi1*B^−^). Unlike the rice transporter, bloom pumpkin transporters were localized in the plasma membrane, while bloomless pumpkin transporters were found in the endoplasmic reticulum. Two efflux Si transporters have also been isolated and characterized in pumpkin [36]. *CmLsi2-1* and *CmLsi2-2* seem to be expressed in both roots and shoots with no sequence disparity. This confirms that if there is a mutation in *CmLsi1*, it may lead to low Si uptake in bloomless pumpkins rather than any mutation in *CmLsi2-1* and *CmLsi2-2*.

**Figure 3:**
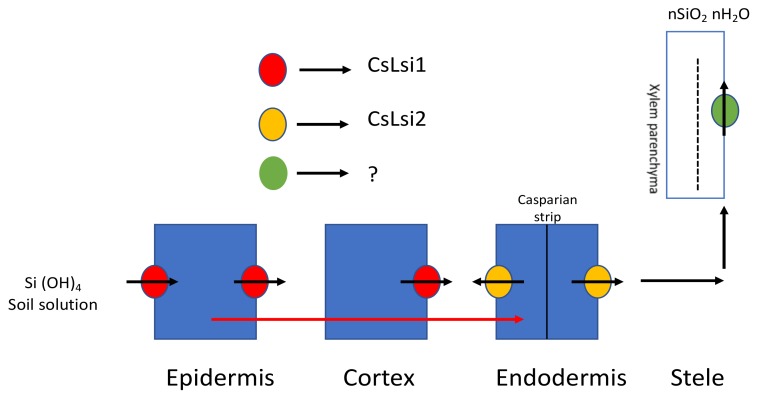
Si uptake and transport in *Cucumis sativus*. *CsLsi1*, the influx transporter, takes up Si from an external solution, and *CsLsi2*, the efflux transporter, further transports it to the endodermis by symplastic pathway. However, the transporter for xylem unloading has not yet been identified. (?—No information is available in literature so far). Modified with reference [32].

Cucumber (*Cucumis sativus*), a commonly grown cucurbitaceous vegetable, accumulates more Si compared with other eudicots. In a study, it was concluded that more than half of the Si taken up by cucumber roots reaches the shoots [18], while, in another study, it was shown that Si uptake is a concentration-independent, active process that follows the Michaelis curve [16]. *CsLsi1*, the Si cucumber influx transporter gene with localization in the distal side of the endodermis and the cortical cells in the root tips, was isolated and characterized in root hairs [37]. *CsLsi1* showed 55.7% and 90.6% homology with rice and pumpkin *Lsi1* transporter genes, respectively. *CsLsi2*, an efflux Si transporter, has also been identified and characterized recently; it is located in the plasma membrane, and its uptake is an energy-dependent process [38]. Transient expression in a *Xenopus laevis* oocyte showed efflux activity for transport of Si. Synchronization of both *CsLsi1* and *CsLsi2* mediates Si uptake and transport across the cell (Figure 3).

*GmNIP2-1* and *GmNIP2-2*, two Si influx transporter genes have been identified and characterized in soybean in a comparative study of a Si-accumulating (rice) and a non-accumulating (Arabidopsis) species [39]. The permeability of these transporters towards Si uptake was tested using *Xenopus* oocyte bioassay. Potato, the third most important crop plant after wheat and rice, shows a potential response to the application of Si fertilizers. A Si transporter homologous with that of pumpkin led to the discovery of a putative Si transporter, *StLsi1*, in potato [40]. *StLsi1* is localized in the roots and leaves; however, its expression rates were much higher in the roots compared with the leaves. 

### 3.2. Cryptogams

*Equisetum arvense* (horsetail) is the highest Si accumulator; however, it has always been neglected, as early efforts by scientists to identify Si transporters proved unsuccessful. Based upon the phylogenetic analysis of its sequence, it was revealed that horsetail belongs to a new multigene family of NIPs, which is a different group compared with that of the already identified plant Si transporters [41]. This newly identified protein group contains a STAR pore in contrast with the previously identified GSGR pore in other known Si transporters. These plants are more inclined, in terms of homology, towards the NIP II group than the NIP III group. The NIP II group comprises of transporters for other small, uncharged molecules. In order to study the transmembrane flux, heterologous protein expressions were carried out in a *Xenopus* oocyte, and it was found that aquaporins from three different horsetail subgroups, EaNIP3;1, EaNIP3;3, and EaNIP3;4, with STAR pores were very efficient Si transporters. Therefore, it was concluded that EaNIP3;1 and EaNIP3;4 were the most efficient, known plant Si transporters, exhibiting even higher activity than rice transporters.

*EaLsi2-1* and *EaLsi2-2*, two putative Si efflux transporter genes, have also been identified in horsetail, showing less than 50% homology to rice gene transporters [42]. In these plants, structural differences with rice *Lsi2* genes can also been attributed to the presence of two introns instead of one. Both these transporter genes are expressed in the roots and shoots, though level of expression were much higher in the shoots than the roots, suggesting that both contribute to Si transport. 

## 4. Conclusions and Future Prospects

Si provides a number of benefits for plants; although it is not regarded as an essential element, it plays an important role against several biotic and abiotic factors. Unfortunately, the majority of plants do not benefit from these effects, as they are either deprived of an efficient Si transport system or the molecular mechanism underlying the uptake and transport of Si in these plants is poorly understood. It is believed that various transporters are involved in the Si uptake mechanism, but only a few could be identified so far. Table 2 shows the presence and absence of Si transporters in different plant families. 

To study the Si uptake and transport mechanism via transporters into the plants at the cellular level, the extraction and characterization of the amino-acid sequence in relation to functional proteins needs to be explored. Moreover, understanding the polar localization of these transporters is another crucial step to identify the directional flow of Si into and outside the cells. The resurgence of interest in studying the Si uptake mechanism through the identification of putative genes and controlled gene expressions will also fast-track this research; as with molecular approaches, specific Si transporter genes can be addressed, whereas others involved in general regulatory functions can be eliminated.

In the current review, we tried to highlight the transport mechanism involved in Si uptake in monocots, eudicots, and few cryptogams. However, in investigating the benefits of Si, it is of utmost importance to study more closely the underlying molecular mechanism of Si uptake in other plant species. Owing to recent developments in this field of research, it is quite clear that many other mechanisms can be explored and understood in greater depth. 

## Figures and Tables

**Table 1:** Different classes of Si accumulators, including high, low, and intermediate accumulator plant species.

Group	Species	Si Accumulation
Angiosperms	Acoraceae *Acorus calamus*	-
	Asteraceae *Anaphalis margarita*	-
	*Helianthus annuus*	+
	*Helianthus maximilianii,*	+
	*Helianthus atrorubens*	+
	*Inula helenium, Inula viscosa*	±
	*Lactuca serriola*	±
	Cyperaceae (*Carex cinica*)	+
	Poaceae *Agrostis spp.*	+
	*Andropogon scoparius*	+
	*Arundinaria gigantean*	+
	*Bouteloua hirsuta*	+
	*Brachypodium sylvaticum*	+
	*Chasmanthium latifolium*	+
	*Ctenium aromaticum*	+
	*Echinochloa colona*	±
	*Elymus molli*	±
	*Oryza sativa*	+
	*Zea mays*	±
	Cucurbitaceae *Benincasa hispida*	±
	*Citrullus lanatus*	±
	*Ecballium elaterium*	±
Equisetophyta	Equisetaceae *Equisetum arvense*	+
	*Equisetum hyemale*	+

+: Si accumulators; ±: Intermediate Si accumulators; -: Low/non-accumulators. Modified with reference [10,11,12].

**Table 2:** The presence of Si transporters in various plant families.

Plant Species	Si Transporters
Angiosperms (monocots and a few eudicots)	+
Equisetopsida	+
Gymnosperms	-
Lycophytes	-
Bryophytes (Liverworts, Mosses, Hornworts)	-
Gnetophytes	-

Modified with reference from Trembath [43].
